# CERMEP-IDB-MRXFDG: a database of 37 normal adult human brain [^18^F]FDG PET, T1 and FLAIR MRI, and CT images available for research

**DOI:** 10.1186/s13550-021-00830-6

**Published:** 2021-09-16

**Authors:** Inés Mérida, Julien Jung, Sandrine Bouvard, Didier Le Bars, Sophie Lancelot, Franck Lavenne, Caroline Bouillot, Jérôme Redouté, Alexander Hammers, Nicolas Costes

**Affiliations:** 1 CERMEP-Imagerie du Vivant, Lyon, France; 2 INSERM U1028/CNRS UMR5292, Lyon Neuroscience Research Center, Lyon, France; 3 Hospices Civils de Lyon, University Hospitals, Lyon, France; 4 Université Claude Bernard Lyon 1, Lyon Neuroscience Research Center, INSERM, CNRS, Lyon, France; 5 School of Biomedical Engineering and Imaging Sciences, Kings’ College London, King’s College London and Guy’s and St Thomas’ PET Centre, London, UK; 6 Neurodis Foundation, Lyon, France; 7 CHU de Lyon HCL - GH Est, 59 Boulevard Pinel., 69677 Bron Cedex, France

**Keywords:** Neuroimaging, PET-FDG, MRI, CT, Healthy subjects, Database sharing

## Abstract

We present a database of cerebral PET FDG and anatomical MRI for 37 normal adult human subjects (CERMEP-IDB-MRXFDG). Thirty-nine participants underwent static [^18^F]FDG PET/CT and MRI, resulting in [^18^F]FDG PET, T1 MPRAGE MRI, FLAIR MRI, and CT images. Two participants were excluded after visual quality control. We describe the acquisition parameters, the image processing pipeline and provide participants’ individual demographics (mean age 38 ± 11.5 years, range 23–65, 20 women). Volumetric analysis of the 37 T1 MRIs showed results in line with the literature. A leave-one-out assessment of the 37 FDG images using Statistical Parametric Mapping (SPM) yielded a low number of false positives after exclusion of artefacts. The database is stored in three different formats, following the BIDS common specification: (1) DICOM (data not processed), (2) NIFTI (multimodal images coregistered to PET subject space), (3) NIFTI normalized (images normalized to MNI space). *Bona fide* researchers can request access to the database via a short form.

**Supplementary Information:**

The online version contains supplementary material available at 10.1186/s13550-021-00830-6.

## Introduction

Imaging databases are very useful to re-analyse data in a different context, to increase the number of subjects of a study, and to develop new methods. Imaging databases play a crucial role in numerous analysis methods that rely in the comparison between the data of a group or of an individual and a group of reference. This includes studies using a normative database for analysis and quantification purposes (such as partial volume correction), machine learning approaches, multi-atlas techniques, and validation of image processing pipelines. Databases with different modalities per participant also allow approaches that derive “missing” modalities, e.g. creating pseudo-CTs for attenuation correction in PET-MR [1–4].

In the last years, an increasing number of neuroimaging databases has been made available. These databases generally consist of MR images (such as ADNI http://adni.loni.usc.edu, OASIS https://www.oasis-brains.org; [5, 6], for a review see [7, 8]). There is also a large database of PET from the Copenhagen group, CIMBI, containing mainly serotonine receptor PET and associated data [9]. We are aware of very few datasets for [^18^F]fluorodeoxyglucose ([^18^F]FDG) PET imaging that have been published [10]) or are available on request ([11]; [12]; Alzheimer’s Disease Neuroimaging Initiative, ADNI http://adni.loni.usc.edu).

Acquisition of imaging data, such as MRI scanning and in particular PET imaging that requires the injection of a radiotracer, represents an important logistical and monetary cost. In addition, participants have to consent to data acquisition and dissemination, and many countries have restrictions on using ionising radiation in healthy controls, adding to difficulties in acquiring such databases. Database sharing thus contributes to reduce research costs and reduces radiation exposure of healthy controls.

In order to make database sharing more efficient, the scientific community has implemented a database standardisation to organize and describe the data (Brain Imaging Data Structure (BIDS), https://bids.neuroimaging.io, [13]) and more specifically for PET modality (https://bids-specification.readthedocs.io/en/bep-009/04-modality-specific-files/09-positron-emission-tomography.html, [14]). In this work we introduce a multi-modal database of 37 healthy subjects constructed with MRI, CT and [^18^F]FDG PET images to BIDS standard. We have obtained ethical permission to share the data on request.

## Materials and methods

### Recruitment and cohort characteristics

All enrolled subjects provided written informed consent to participate in the study (EudraCT: 2014-000610-56). The subjects were informed that their anonymized images could be used for methodological development and had been given the option to oppose this use of their data. The inclusion criteria were adult healthy subject and aged between 20 and 65 years. Exclusion criteria were (1) children and adults older than 65 years, (2) woman of childbearing potential without effective contraception, (3) history of neurological disorders, (4) any contraindication for MRI scanning, (5) active infectious disease. Thirty-nine subjects were included in the study. Each subject had a T1-weighted MRI, a T2 fluid-attenuated inversion recovery (FLAIR) MRI and an [^18^F]FDG PET/CT brain scan. For all participants, the PET/CT scan and the MRI session took place on the same day (between 8 a.m. and 14 p.m.). The subjects’ MR and PET images were visually reviewed by two neurologists for conspicuous brain abnormalities. Two subjects showing brain lesions on the MR images (one probable insular cavernoma, one cerebellar lesion with hyperintense signal in the FLAIR sequence suggesting possible inflammatory disease of the central nervous system) were excluded from the database.

### MRI acquisition and reconstruction

MRI sequences were obtained on a Siemens Sonata 1.5 T scanner. Three-dimensional anatomical T1-weighted sequences (MPRAGE) were acquired in sagittal orientation (TR 2400 ms, TE 3.55 ms, inversion time 1000 ms, flip angle 8°). The images were reconstructed into a 160 × 192 × 192 matrix with voxel dimensions of 1.2 × 1.2 × 1.2 mm^3^ (axial field of view 230.4 mm). Sagittal Fluid-Attenuated Inversion Recovery (FLAIR, [15]) images (TR 6000 ms, TE 354 ms, Inversion time 2200 ms, flip angle 180°) were acquired with a 176 × 196 × 256 matrix and a voxel size of 1.2 × 1.2 × 1.2 mm^3^ (axial field of view 307.2 mm).

### PET and CT acquisition and reconstruction

PET and CT data were acquired on a Siemens Biograph mCT64. During the uptake period, participants were instructed to rest with their eyes closed and without auditory stimulation. A static PET data acquisition started 50 min after the injection of 122.30 ± 21.29 MBq of [^18^F]FDG (individual doses are provided in the demographics table) and lasted 10 min [16]. PET images were reconstructed using 3D ordinary Poisson-ordered subsets expectation maximization (OP-OSEM 3D), incorporating the system point spread function and time of flight, and using 12 iterations and 21 subsets (Siemens’ “HD reconstruction”). Data correction (normalization, attenuation and scatter correction) was fully integrated within the reconstruction process. Gaussian post-reconstruction 3D filtering (FWHM = 4 mm isotropic) was applied to all PET images [17]. Reconstructions were performed with a zoom of 2 yielding a voxel size of 2.04 × 2.04 × 2.03 mm^3^ in a matrix of 200 × 200 × 109 voxels (axial field of view 221.27 mm). Low-dose CT images for attenuation correction were acquired with a tube voltage of 100 keV and reconstructed in a 512 × 512 × 233 matrix with a voxel size of 0.6 × 0.6 × 1.5 mm^3^ (axial field of view 349.5 mm).

### Processing pipeline

#### Data anonymisation and pre-processing

Data anonymisation was performed on the DICOM files using the *gdcmanon* function (http://gdcm.sourceforge.net/html/gdcmanon.html). DICOM files were converted to NIFTI format with *dcm2niix* software (https://github.com/rordenlab/dcm2nii). The background of CT images was cleaned in order to remove the scanner table and other objects such as the pillow included in the background of the image. For this, a binary mask of the head of the subject was automatically generated following a procedure described in [18] using tools from the FSL (Version 6.0, https://fsl.fmrib.ox.ac.uk/fsl/fslwiki/) and NiftySeg (http://cmictig.cs.ucl.ac.uk/wiki/index.php/NiftySeg) suites. Finally, the binary mask was applied to the CT image.

#### Coregistration

As first step, the origin of each NIFTI image was set to the matrix centre. Then, CT, T1 MRI and FLAIR MRI images were coregistered to the [^18^F]FDG PET image using the *Coregister & Estimate* function from the SPM 12 toolbox (https://www.fil.ion.ucl.ac.uk/spm/software/spm12/).

#### Spatial normalisation

All images were normalized to MNI space through the tissue classification into grey and white matter probability maps of the T1 image. For that, individual subject’s deformation fields were calculated by the *Segment* function of SPM 12 [19] from the T1 images previously coregistered to the PET image (but not resliced to preserve native resolution). Transformations for MR to PET space coregistration and PET to MNI space normalisation were concatenated and applied at once to avoid an intermediate resampling of the MRI data. All normalized images were resampled at 1 × 1x1 mm using 4th degree B-spline interpolation.

#### Intensity normalisation

Reconstructed PET images were normalized by the subjects’ weight and injected dose to obtain Standard Uptake Value (SUV) images (radioactivity concentration [kBq/cm^3^] / (dose [kBq] / weight [kg])). In addition, reconstructed PET images were normalized by each subject’s mean activity within the intracranial volume (ICV) mask provided by SPM12 to obtain Standard Uptake Value ratio (SUVr).

### Regional analysis

The T1 MR images were anatomically segmented into 83 regions using the Hammers_mith maximum probability atlas n30r83, which is based on the mutli-atlas fusion of 30 manually delineated MRIs of healthy young adults [5, 6], available at http://brain-development.org. The atlas was wrapped to each individual MRI space via the inverse transformation of the deformation fields from subject’s space to the MNI space computed at the spatial normalisation step. Grey matter and white matter probability maps obtained with the *Segment* function were thresholded at 0.5 and combined with the 83-ROI anatomical segmentation in order to separate their grey and white matter parts, expect for pure white matter regions like the corpus callosum, and pure grey matter regions like the basal ganglia. Mean regional SUV and SUVr were extracted in a selection of grey matter anatomical regions of the Hammers_mith segmentation.

### Leave-one-out SPM analysis on [^18^F]FDG images

Leave-one-out ANCOVA was performed on SPM12 in order to compare each subject (healthy control) of the database to the others. For the statistical analysis, PET images were smoothed with a Gaussian filter at 8 mm FWHM. This further smoothing is always used in voxel-based analysis to accommodate interindividual anatomical variability and improve the sensitivity of the statistical analysis [20]. We used age and the global mean calculated within the intracranial volume mask as covariates. Two different contrasts were explored: Hyper-metabolism, i.e. activity of one subject > activity of the remaining subjects in the database, and hypo-metabolism, i.e. activity of one subject < activity of the remaining subjects in the database. Significant differences where defined at p < 0.05 FWE at the cluster level.

The database outliers were assessed with three criteria, for both hypometabolism and hypermetabolism.

- Subject-level: number of subjects with significant differences / total number of subjects in the database × 100
- Cluster-level: total number of significant clusters across all subjects / average number of resolution elements (resells) in the mask × 100
- Voxel-level: total number of voxels among the significant clusters across all subjects / number of voxels in the SPM mask × 100

## Results

### Database IDB-MRXFDG

**Fig. 1:**
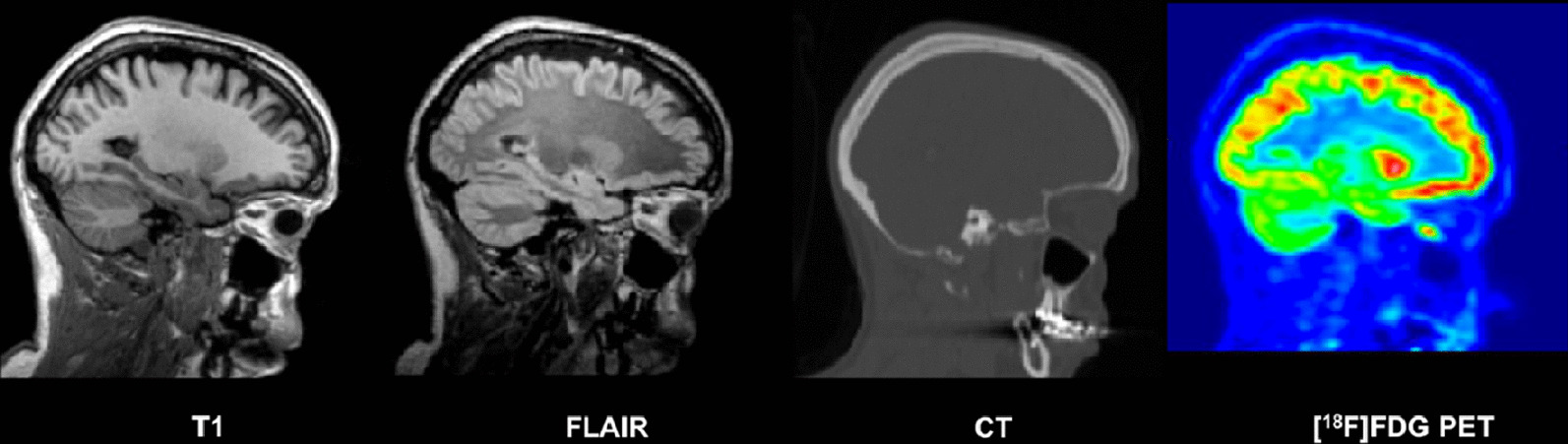
Example of coregistered T1 MRI, FLAIR MRI, CT and [^18^F]FDG PET images (sagittal plane) for one subject of the database

**Fig. 2:**
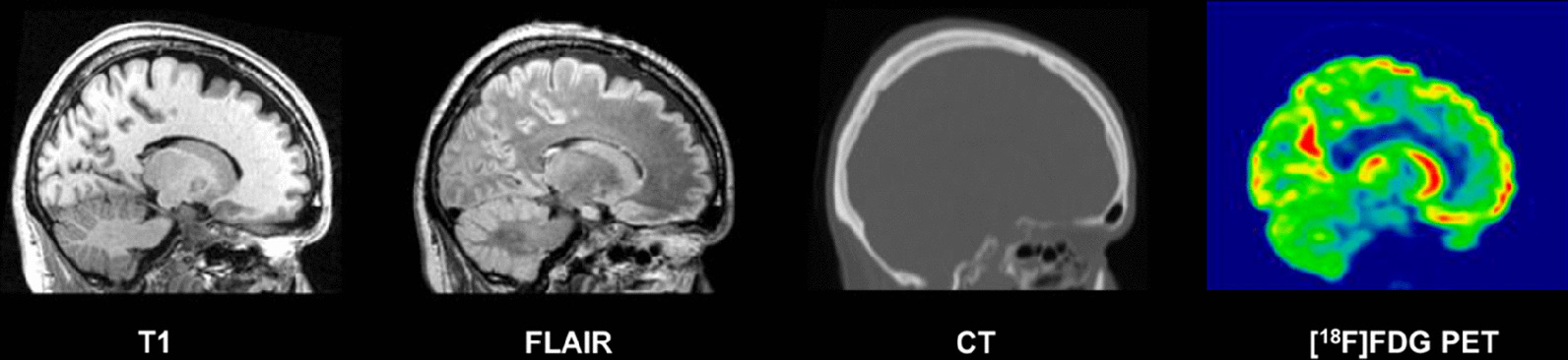
Example of normalized T1 MRI, FLAIR MRI, CT and [^18^F]FDG PET images (sagittal plane) in MNI space, for one subject of the database

The final database consists of 37 participants (17 male/20 female, mean age ± SD, 38.11 ± 11.36 years; range, 23–65 years). Each participant has [^18^F]FDG PET, T1 MRI, FLAIR MRI, and CT images. An example of coregistered T1, FLAIR, CT and [^18^F]FDG PET images in the subject space are shown in Fig. 1 and the same images in normalized space are shown in Fig. 2. Table 1 summarizes the demographic information for each participant: subject ID, acquisition date, age of the participant at the time of the imaging session, sex, weight, size, injected dose of [^18^F]FDG, handedness and a comment if any hypersignal was observed on the FLAIR MRI.

**Table 1:** Demographics table

Subject ID	Birth date (year)	Age	Sex	Weight (kg)	Size (cm)	Injected dose (MBq)	Mean activity in ICV mask (MBq)	Handedness
sub-0001	1980	35	F	81	163	149	6911	R
sub-0002	1957	58	F	66	160	102	5781	R
sub-0003	1979	36	M	88	174	144	5980	R
sub-0004	1963	51	M	72	–	124	6551	R
sub-0005	1981	33	M	110	180	147	4925	R
sub-0006	1974	41	M	76	176	119	5590	R
sub-0007	1975	40	F	60	170	118	8271	R
sub-0008	1988	27	F	53	–	95	6889	R
sub-0009	1986	28	F	66	–	135	7974	R
sub-0010	1971	43	M	117	170	168	6137	R
sub-0011	1988	27	F	53	161	99	6944	R
sub-0012	1990	24	F	63	168	123	7911	R
sub-0013	1988	27	M	74	178	135	7798	R
sub-0014(*)	1964	50	F	55	157	94	2574	R
sub-0015	1981	34	M	62	170	109	6032	R
sub-0016(*)	1949	65	F	70	170	108	4849	R
sub-0017	1958	56	M	89	185	140	5539	L
sub-0018	1969	45	M	71	168	132	4444	R
sub-0019	1989	25	F	61	161	109	7755	R
sub-0020	1973	42	M	72	185	135	6966	L
sub-0021	1990	25	M	63	178	110	3119	R
sub-0022	1990	25	M	72	178	133	7075	R
sub-0023	1974	41	F	54	168	99	5970	R
sub-0024	1976	39	M	118	199	167	6528	R
sub-0025(*)	1967	48	F	83	168	149	5811	R
sub-0026	1988	27	F	70	–	134	7656	R
sub-0027	1963	52	F	57	–	107	6647	R
sub-0028	1969	46	F	53	173	95	5481	L
sub-0029	1991	23	M	80	178	136	7824	R
sub-0030	1989	25	M	72	175	124	7384	R
sub-0031	1979	35	F	58	–	112	6621	R
sub-0032	1981	34	M	80	178	136	7011	R
sub-0033	1955	60	F	48	159	95	6151	R
sub-0034	1984	31	F	58	166	106	6119	L
sub-0035	1969	46	F	48	167	96	6066	R
sub-0036	1982	33	M	79	180	149	7253	R
sub-0037	1981	33	F	47	163	92	7355	R
Mean		38.11		70.24	171.81	122	6375	
SD		11.49		17.37	9.11	21	1276	
Min		23		48	157	94	2574	
Max		65		118	199	168	8271	

(*)sub-0014: Hyperintense FLAIR signals in white matter (corona radiata) suggesting benign age related white matter hyperintensities (WMHs)

sub-0016: Hyperintense FLAIR signals in white matter (corona radiata and frontal subcortical structures) suggesting benign age related white matter hyperintensities

sub-0025: Hyperintense FLAIR signals in white matter (corona radiata and frontal subcortical structures) suggesting benign age related white matter hyperintensities

For those 3 subjects, the location and MRI changes observed in FLAIR sequences are typical findings of WMHs with diffuse areas of high signal intensity (hence, “hyperintense”) on T2-weighted or FLAIR sequences. Those WMHs are typically interpreted as a surrogate of cerebral small vessel disease. Due to the high prevalence of those MRI changes in asymptomatic subjects above 50 years, the PET images of those subjects were included in the database

The database is available in three different formats, following the BIDS common specification:

- DICOM (data not processed)
- NIFTI (multimodal images coregistered to PET subject space)
- NIFTI normalized (images normalized to MNI space)

Table 2 lists the regional volumes obtained via the Hammers_mith maximum probability atlas. Coefficients of variation were as expected, without obvious outliers. The structure sizes were also in line with expectations [5, 6].

**Table 2:** Regional volumes in native space (in cm^3^)

Structure name	Grey matter	White matter	Structure name	Grey matter	White matter
	Mean ± SD	Mean ± SD		Mean ± SD	Mean ± SD
**Temporal Lobe**			**Occipital Lobe**		
Hippocampus_r	2.37 ± 0.28	–	OL_rest_lat_l	21.95 ± 2.40	18.27 ± 2.83
Hippocampus_l	2.11 ± 0.26	–	OL_rest_lat_r	22.42 ± 2.54	19.36 ± 3.19
Amygdala_r	1.43 ± 0.17	–	OL_ling_G_l	7.56 ± 0.93	3.94 ± 0.70
Amygdala_l	1.52 ± 0.18	–	OL_ling_G_r	8.16 ± 0.91	3.97 ± 0.79
Ant_TL_med_r	5.48 ± 0.64	1.24 ± 0.28	OL_cuneus_l	5.31 ± 0.66	3.34 ± 0.61
Ant_TL_med_l	5.27 ± 0.61	1.22 ± 0.26	OL_cuneus_r	5.72 ± 0.70	3.09 ± 0.67
Ant_TL_inf_lat_r	2.77 ± 0.39	0.55 ± 0.19	**Parietal Lobe**		
Ant_TL_inf_lat_l	2.58 ± 0.41	0.49 ± 0.17	PL_rest_l	21.37 ± 2.52	16.01 ± 2.40
G_paraH_amb_r	3.15 ± 0.37	0.98 ± 0.13	PL_rest_r	21.25 ± 2.48	15.95 ± 2.28
G_paraH_amb_l	3.27 ± 0.42	0.99 ± 0.18	PL_postce_G_l	11.69 ± 1.58	14.69 ± 1.96
G_sup_temp_cent_r	7.62 ± 0.93	5.33 ± 0.79	PL_postce_G_r	10.80 ± 1.43	13.82 ± 1.76
G_sup_temp_cent_l	7.64 ± 0.96	5.26 ± 0.80	PL_sup_pa_G_l	19.50 ± 2.10	17.65 ± 2.75
G_tem_midin_r	11.24 ± 1.33	5.95 ± 0.95	PL_sup_pa_G_r	20.51 ± 2.15	18.42 ± 3.05
G_tem_midin_l	10.78 ± 1.30	5.87 ± 1.03	**Central Structures**		
G_occtem_la_r	3.42 ± 0.39	0.92 ± 0.24	CaudateNucl_l	4.24 ± 0.52	–
G_occtem_la_l	3.41 ± 0.41	0.94 ± 0.24	CaudateNucl_r	4.33 ± 0.54	–
PosteriorTL_l	26.41 ± 3.01	17.13 ± 2.44	NuclAccumb_l	0.36 ± 0.05	–
PosteriorTL_r	27.52 ± 3.05	17.48 ± 2.39	NuclAccumb_r	0.30 ± 0.04	–
G_sup_temp_ant_l	3.28 ± 0.42	0.67 ± 0.19	Putamen_l	4.92 ± 0.57	–
G_sup_temp_ant_r	3.19 ± 0.39	0.62 ± 0.17	Putamen_r	4.76 ± 0.54	–
**Posterior Fossa**			Thalamus_l	7.41 ± 0.87	–
Cerebellum_r	44.49 ± 5.10	12.34 ± 1.63	Thalamus_r	7.25 ± 0.86	–
Cerebellum_l	44.36 ± 5.13	12.39 ± 1.63	Pallidum_l	1.30 ± 0.16	–
Brainstem	23.05 ± 2.82	–	Pallidum_r	1.31 ± 0.16	–
**Frontal Lobe**			Corp_Callosum	20.83 ± 2.54	–
FL_mid_fr_G_l	23.21 ± 3.01	24.36 ± 3.67	S_nigra_l	0.32 ± 0.04	–
FL_mid_fr_G_r	23.59 ± 2.95	24.52 ± 3.57	S_nigra_r	0.32 ± 0.04	–
FL_precen_G_l	13.11 ± 1.76	18.47 ± 2.32	**Ventricles**		
FL_precen_G_r	13.04 ± 1.84	18.55 ± 2.37	BodyVentricle_r	7.31 ± 1.01	–
FL_OFC_AOG_l	3.87 ± 0.51	1.45 ± 0.39	BodyVentricle_l	7.98 ± 1.01	–
FL_OFC_AOG_r	3.89 ± 0.51	1.45 ± 0.35	TemporaHorn_r	0.63 ± 0.08	–
FL_inf_fr_G_l	10.63 ± 1.34	5.96 ± 1.12	TemporaHorn_l	0.49 ± 0.06	–
FL_inf_fr_G_r	10.15 ± 1.24	5.50 ± 1.05	ThirdVentricl	0.94 ± 0.13	–
FL_sup_fr_G_l	27.15 ± 3.58	20.00 ± 3.10	**Insula and Cingulate gyri**		
FL_sup_fr_G_r	27.33 ± 3.63	19.71 ± 3.12	Insula_l	15.42 ± 1.77	–
FL_OFC_MOG_l	3.95 ± 0.53	1.83 ± 0.37	Insula_r	15.37 ± 1.79	–
FL_OFC_MOG_r	3.93 ± 0.48	1.63 ± 0.30	G_cing_ant_sup_l	5.56 ± 0.74	1.66 ± 0.43
FL_OFC_LOG_l	2.13 ± 0.31	0.92 ± 0.22	G_cing_ant_sup_r	5.29 ± 0.69	1.67 ± 0.40
FL_OFC_LOG_r	2.39 ± 0.37	0.91 ± 0.24	G_cing_post_l	5.07 ± 0.67	2.52 ± 0.46
FL_OFC_POG_l	3.14 ± 0.41	1.34 ± 0.28	G_cing_post_r	4.95 ± 0.60	2.36 ± 0.43
FL_OFC_POG_r	3.27 ± 0.38	1.20 ± 0.27			
FL_strai_G_l	2.69 ± 0.34	0.60 ± 0.13			
FL_strai_G_r	2.88 ± 0.34	0.77 ± 0.20			
Subgen_antCing_l	0.78 ± 0.13	0.59 ± 0.10			
Subgen_antCing_r	0.69 ± 0.13	0.54 ± 0.10			
Subcall_area_l	0.21 ± 0.04	0.02 ± 0.01			
Subcall_area_r	0.18 ± 0.03	0.02 ± 0.01			
Presubgen_antCing_l	0.76 ± 0.11	0.11 ± 0.05			
Presubgen_antCing_r	0.50 ± 0.07	0.08 ± 0.05			

Each paired region is composed of left and right sub-regions. The short names are expanded in Additional file 1: Table S3

### Regional analysis

**Fig. 3:**
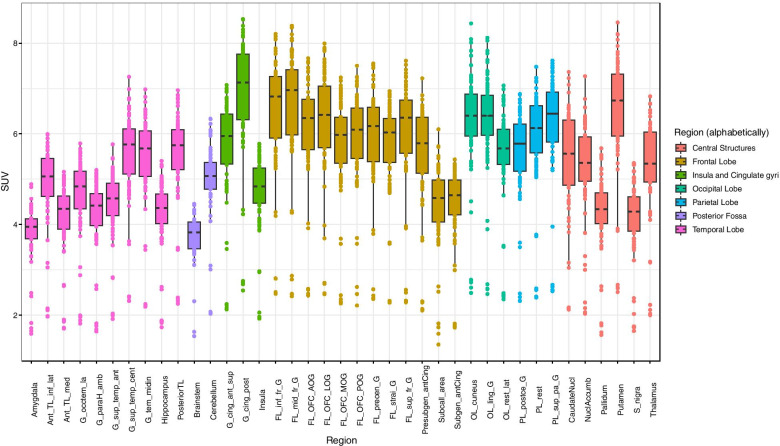
Boxplot of regional SUV for all subjects in the database. Centre lines correspond to medians, boxes to interquartile ranges, and whiskers to robust ranges. Outliers are represented as dots. Each dot represents a participant for unpaired regions and a participant’s right or left SUV value for paired regions

**Fig. 4:**
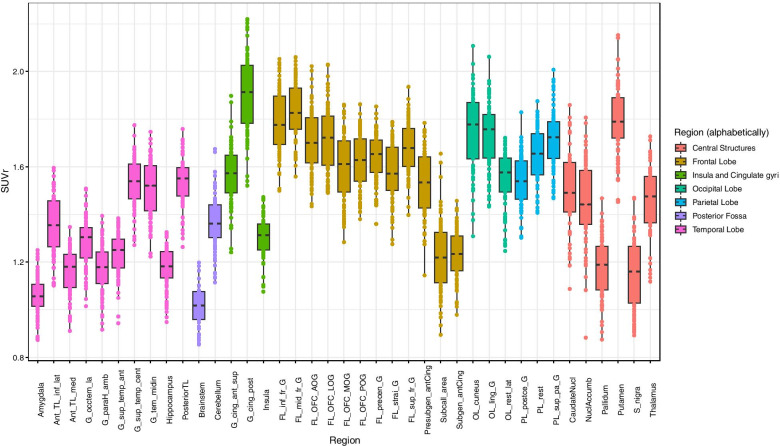
Boxplot pf regional activity normalized by mean activity in ICV mask (SPM) for all subjects in the database. Centre lines correspond to medians, boxes to interquartile ranges, and whiskers to robust ranges. Outliers are represented as dots

Figures 3 and 4 show boxplots of mean regional SUV and SUVr respectively, extracted in a selection of grey matter anatomical regions, for all subjects in the database. Each region is composed of left and right sub-regions. Mean regional SUV values were 5.36 ± 1.32, range 1.35–8.54 (Fig. 3). Three subjects in the database had lower SUV values (between 1.35 and 3). The distribution of SUVr values (Fig. 4) remains very similar to the distribution of SUV values (1.49 mean ± 0.26 SD, range 0.85–2.22), except that the dispersion is reduced and the outlier values from the three participants with unusually low SUVs are regularized. Normalizing with the ICV mean value thus acts as an efficient way for regularizing the SUV distribution leaving the inter-regional variability intact.

### Leave-one-out SPM analysis

Results for the leave-one-out analysis of [^18^F]FDG PET are reported in Table 3. At the subject-level, 5/37 (13.5%) of the participants had any significant increases in [^18^F]FDG uptake (hypermetabolism) relative to the other 36 participants. Any significant decreases (hypometabolism) was found for 11/37 (29.7%) of the participants.

**Table 3:** % of abnormality in the database

Contrast	Subject-level	Cluster-level (%)	Voxel-level (%)
Hypermetabolism	13.5% (*n* = 5)	0.93	0.03
Hypometabolism	29.7% (*n* = 11)	5.21	0.32

Cluster-level and voxel-level results are reported at *p* < 0.05 FWE. The denominator for subject-level is the total number of participants; the denominator for the cluster-level is the average number of resolution elements in the mask; the denominator for the voxel-level is the number of voxels in the SPM mask. See Methods for details

At the cluster-level, significant changes were found in at most 5.21% of resolution elements, and at the voxel-level, in at most 0.32% of voxels. All abnormalities in controls compared with controls are by definition false positives. We examined all 16 and present our findings in the Additional file 1 (Table S1 and Table S2). Virtually all false positives had an anatomical or artefactual explanation.

## Discussion

A new database of 37 healthy subjects including T1 and FLAIR MRI, CT, and [^18^F]FDG PET images, called IDB-MRXFDG, has been created.

The age range has been selected to reflect the ages of participants in cognitive and clinical research studies at the CERMEP imaging centre, encompassing amongst others epilepsy, movement disorders, multiple sclerosis and disorders of consciousness and will align with the research priorities of many similar centres.

We performed quality control of all images visually and by screening for volumetric and regional SUV abnormalities. Three subjects had unusually low SUVs; this may be due to imperfect observation of the need for fasting ahead of the scan. This could have been ascertained by measuring the blood glucose level which was not measured here, which is a limitation of the study. We show that a simple global normalisation procedure removes the resulting outliers (Fig. 4); depending on the application more sophisticated intra-scan normalisation procedures are conceivable [21, 22]. We also performed SPM leave-one-out studies for [^18^F]FDG. The relatively high false-positive rates per subject are explained by the existence of significant clusters of small size (from 1 to 95 voxels). Areas of apparent hypermetabolism were either at the edge of the brain or at the bottom of a particularly deep sulcus (see Additional file 1: Table S1); areas of apparent hypometabolism (Additional file 1: Table S2) were clearly linked to the participant’s anatomy, typically to a wide sulcus or fissure (7/11 cases). The other 4 cases were extracerebral or at the edge of the brain, probably linked to imperfect normalisation. We believe none would have been considered abnormal had they been seen in an analysis comparing one research subject with a particular condition against a group of controls. When testing the normality of the database at the cluster and voxel-level, the expected threshold of 5% of abnormality or lower was found for both hyper- and hypo-metabolism. The database therefore appears suitable for voxel-based [^18^F]FDG PET analysis with a ≤ 5% risk of Type 1 error.

The IDB-MRXFDG database could be used in many different applications such as the statistical comparison of a patient (or group of patients) to a database of healthy subjects, automatic quantitative analyses, and more generally methodology development in neuroimaging.

The inclusion of [^18^F]FDG PET in IDB-MRXFDG is particularly important. While there are now many MR databases covering, with varying density, the human lifespan as reviewed in [7], we are aware of very few [^18^F]FDG PET databases. Wei et al. [10]) scanned 78 healthy subjects aged 3–78 years on a PET/CT scanner; it is not clear whether this database is available on request, and there is no mention of MRI. The Marseille database (used e.g. in [12]) contains data from 60 healthy adults aged 21–78; [^18^F]FDG PET, T1 weighted MRI, and CT data are available by arrangement. A rare paediatric database [11] contains 24 datasets of participants aged 4.5–17.9 years (mean ± SD 10.06 ± 3.1 years) and may be shared on request. These are “pseudo-controls” derived from epilepsy patients, selected from among a total of 71 children as the subgroup with both a normal visual analysis and a normal SPM analysis derived iteratively. They have been scanned on a traditional PET scanner with transmission-based attenuation correction which makes comparison with PET/CT data difficult [23]; no MRI is available. A large database available on request is the Alzheimer’s Disease Neuroimaging Initiative, ADNI (http://adni.loni.usc.edu/about/) which comprises over 300 healthy control [^18^F]FDG PET datasets; however, participants are aged 55–90 and therefore more suited to dementia research but outside the typical age range used for studies in normal cognition or epilepsy, one of the main clinical indications for brain FDG PET. Similar concerns about the age of participants apply to those databases from the world-wide ADNI (WW-ADNI) networks that do contain FDG, as for example the Japan ADNI (J-ADNI; age 60–84) [24].

It should be noted that we used very high-quality reconstructions incorporating both the system point spread function and time-of-flight information, which will not be available on all machines. If lower resolution images are required, the images could simply be filtered with a gaussian kernel (e.g. [25]).

Examples of database uses for work in **MR** include the *voxel-wise* comparison of a patient with a control group to detect abnormalities from T1 images via voxel-based morphometry [26, 27] and its variants that use T1 derivatives like grey-white matter junction images [28, 29] for the detection of specific pathologies like Focal Cortical Dysplasia. FLAIR as a sequence highly sensitive to pathology has similarly been used at the single-subject level in comparison to control groups (e.g. [30, 31]). Another group of examples is the *region-wise* comparison of the size of cerebral structures between groups or between individuals and a control group (e.g. [32–35]). Importantly, such work has been successfully undertaken with control groups scanned on a different scanner (e.g. [36, 37]), and IDB-MRXFDG could be used to increase the size of control groups.

The multimodality aspect of IDB-MRXFDG is particularly important.

Since PET-CT scanners rapidly displaced PET-only scanners in the early 2000s, low-dose CT has been coupled to brain [^18^F]FDG PET for estimation of tissue density and attenuation correction. With the advent of commercial PET-MR scanners since 2011, there has been no direct way of measuring electron density in the head, and alternative approaches have had to be found. Synthesis of “pseudo-CTs” via atlas approaches [1, 2] is a successful approach that performs well overall [38] but requires pairs of MR and CT images to achieve the synthesis. IDB-MRXFDG has already been used for such approaches [39].

The latter application of databases—MR-based attenuation based on MR-CT pairs—is one domain where Deep Learning methods, notably with Convolutional Neuronal Networks, have recently become very successful [3, 40]. However, they often require substantially larger training datasets or priors than multi-atlas methods, in the case of MR-based attenuation recently estimated at 100–400 pairs, with an influence of MR heterogeneity [40]. More widespread availability of databases will further Deep Learning approaches, particularly when multiple modalities are available per subject, allowing e.g. synthesis of missing modalities [41].

Pairs of data are also required for partial volume effect correction methods incorporating structural MRI information (PET-MR pairs; e.g. [42]). The additional availability of FLAIR-T1 pairs can be exploited e.g. for detection of focal cortical dysplasias as the underlying substrate of medically refractory focal epilepsies [30].

## Supplementary Information

**Additional file 1.** Supplemental materials.

## Data Availability

The datasets generated during and/or analysed during the current study are available from the corresponding author on reasonable request.

## Declarations

### Data sharing

We have obtained Ethical permission to make the database available on request for *bona fide* research. Please email (merida@cermep.fr) for a short access form, detailing which format you require (DICOM format, NIFTI in subject’s space and NIFIT in normalized space, following BIDS common specification). The request will then be considered by the access committee. If found in line with permitted use (i.e. *bona fide* research) a licence will be issued and the requested database transferred securely. Please note it cannot be licenced for clinical use.
