# Supplementary material for: CERMEP-IDB-MRXFDG: a database of 37 normal adult human brain [18F]FDG PET, T1 and FLAIR MRI, and CT images available for research
Source: EJNMMI Res. 2021 Sep 16;11:91. doi: 10.1186/s13550-021-00830-6 (PMC8446124; doi:10.1186/s13550-021-00830-6)
Supplement: Supplementary file 1 — Additional file 1. Supplemental materials. [file 13550_2021_830_MOESM1_ESM.pdf]

CERMEP-IDB-MRXFDG: A database of 37 normal adult human brain [18F]FDG PET, T1 and FLAIR MRI, and CT images available for research

Inés Mérida[1*§], Julien Jung[2,6*], Sandrine Bouvard[3], Didier Le Bars[1,6], Sophie Lancelot[1,2,6], Franck Lavenne[1], Caroline Bouillot[1], Jérôme Redouté[1], Alexander Hammers[4,5*], Nicolas Costes[1*]

*Equal contribution

[1]*CERMEP-Imagerie du vivant, Lyon, France*

[2]*INSERM U1028/CNRS UMR5292, Lyon Neuroscience Research Center, Lyon, France*

[3]*Université Claude Bernard Lyon 1, Lyon Neuroscience Research Center, INSERM, CNRS, France*

[4]*King's College London & Guy's and St Thomas' PET Centre, School of Biomedical Engineering and Imaging Sciences, Kings' College London, London, United Kingdom*

[5]*Neurodis Foundation, Lyon, France*

[6]*Hospices Civils de Lyon, University Hospitals, Lyon France*

[§]*Corresponding author*

Supplemental material

*Table S1: Individual thresholded T-maps ($p < 0.05$ FWE) for participants showing significant **increases** in [^{18}F]FDG uptake (hypermetabolism) relative to the other 36 participants (false positives). The analysis consisted in a leave-one-out ANCOVA performed on SPM12 (see Methods for details). For each case, we provide an anatomical or artefactual explanation.*

Participant ID	Comment	Illustration
sub-0002	Normalisation artifact (linear artefact seen when lowering threshold)	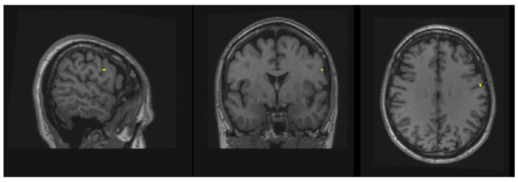
sub-0003	Edge of brain	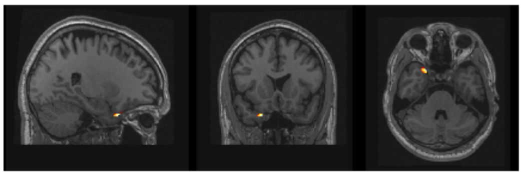
sub-0010	Extracerebral cluster	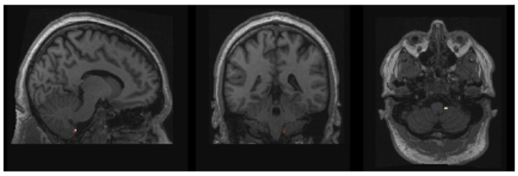
sub-0020	Hypermetabolism due to particular anatomy (grey matter / deep sulcus in this participant, white matter in others)	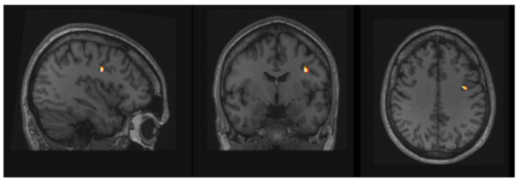
sub-0024	Hypermetabolism due to particular anatomy (grey matter / deep sulcus in this participant, white matter in others)	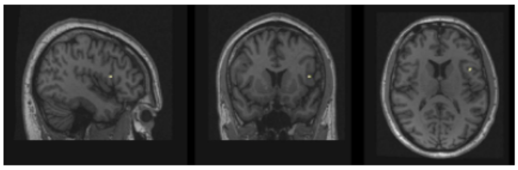

Table S2: Individual thresholded T-maps (p < 0.05 FWE) for participants showing significant ***decreases*** *in [^{18}F]FDG uptake (hypometabolism) relative to the other 36 participants (false positives). The analysis consisted in a leave-one-out ANCOVA performed on SPM12 (see Methods for details). For each case, we provide an anatomical or artefactual explanation.*

Participant ID	Comment	Illustration
sub-0002	Apparent hypometabolism in deep / wide sulcus in this participant	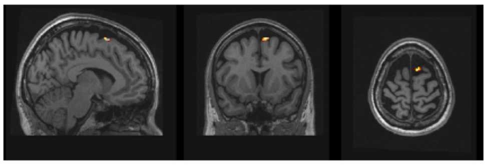
sub-0004	Apparent hypometabolism in deep / wide sulcus in this participant	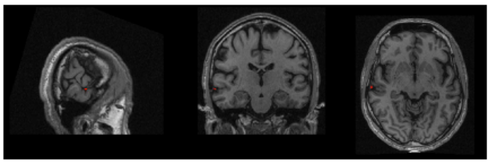
sub-0005	Apparent hypometabolism in deep / wide sulcus in this participant / linear normalisation artefact	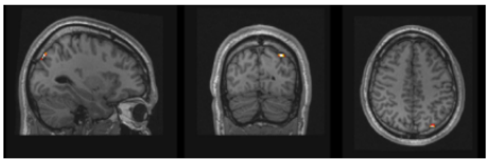
sub-0007	Apparent hypometabolism in deep / wide sulcus in this participant	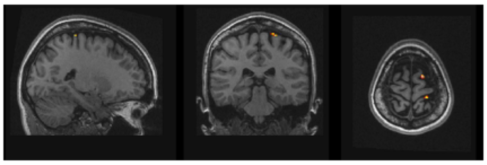
sub-0010	Extracerebral cluster	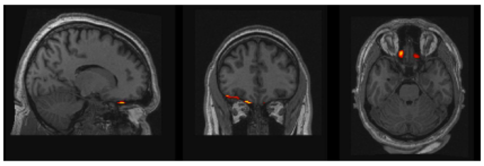
sub-0016	Edge of brain	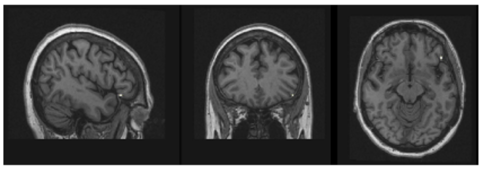
sub-0017	Apparent hypometabolism in deep / wide sulcus in this participant: Typical artifact in wide Sylvian fissure	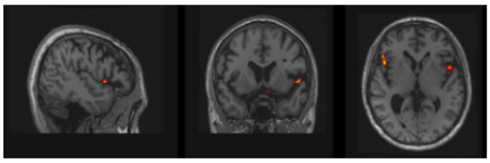
sub-0020	Linear normalisation artefact at edge of brain	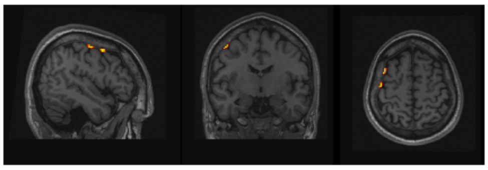

Participant ID	Comment	Illustration
sub-0023	Apparent hypometabolism in unusually wide cerebellar fissure in this participant	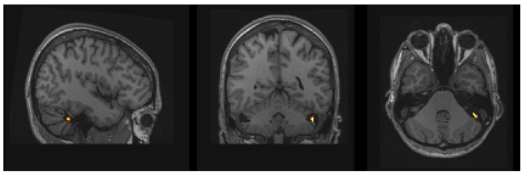
sub-0024	Border effect, possibly linked to imperfect spatial normalisation due to prominent frontal sinuses	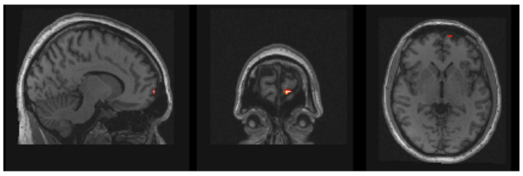
sub-0035	Apparent hypometabolism in deep / wide sulcus in this participant	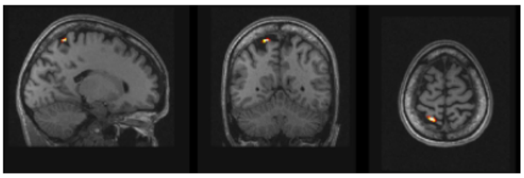

Table S3: Abbreviation list of the 83 regions used in the ROI evaluation based on the Hammers_mith atlases (www.brain-development.org / Hammers et al. 2003, Gousias et al. 2008)

Abbreviation	Complete name
Hippocampus_r	Hippocampus (right)
Hippocampus_l	Hippocampus (left)
Amygdala_r	Amygdala (right)
Amygdala_l	Amygdala (left)
Ant_TL_med_r	Anterior temporal lobe, medial part (right)
Ant_TL_med_l	Anterior temporal lobe, medial part (left)
Ant_TL_inf_lat_r	Anterior temporal lobe, lateral part excluding superior temporal gyrus (right)
Ant_TL_inf_lat_l	Anterior temporal lobe, lateral part excluding superior temporal gyrus (left)
G_paraH_amb_r	Parahippocampal and ambient gyri (right)
G_paraH_amb_l	Parahippocampal and ambient gyri (left)
G_s_t_cent_r	Superior temporal gyrus, central part (right)
G_s_t_cent_l	Superior temporal gyrus, central part (left)
G_tem_midin_r	Middle and inferior temporal gyrus (right)
G_tem_midin_l	Middle and inferior temporal gyrus (left)
G_occtem_la_r	Fusiform (lateral occipitotemporal) gyrus (right)
G_occtem_la_l	Fusiform (lateral occipitotemporal) gyrus (left)
Cerebellum_r	Cerebellum (right)
Cerebellum_l	Cerebellum (left)
Brainstem	Brainstem
Insula_l	Insula (left)
Insula_r	Insula (right)
OL_rest_lat_l	Lateral remainder of occipital lobe (left)
OL_rest_lat_r	Lateral remainder of occipital lobe (right)
G_cing_a_s_l	Cingulate gyrus, anterior part (left)
G_cing_a_s_r	Cingulate gyrus, anterior part (right)
G_cing_p_l	Gyrus cinguli, posterior part (left)
G_cing_p_r	Gyrus cinguli, posterior part (right)
FL_mid_fr_G_l	Middlle frontal gyrus (left)
FL_mid_fr_G_r	Middlle frontal gyrus (right)
PosteriorTL_l	Posterior temporal lobe (left)
PosteriorTL_r	Posterior temporal lobe (right)
PL_rest_l	Inferiolateral remainder of parietal lobe (left)
PL_rest_r	Inferiolateral remainder of parietal lobe (right)
CaudateNucl_l	Caudate nucleus (left)
CaudateNucl_r	Caudate nucleus (right)
NuclAccumb_l	Nucleus accumbens (left)
NuclAccumb_r	Nucleus accumbens (right)
Putamen_l	Putamen (left)
Putamen_r	Putamen (right)
Thalamus_l	Thalamus (left)
Thalamus_r	Thalamus (right)
Pallidum_l	Pallidum (left)
Pallidum_r	Pallidum (right)
Corp_Callosum	Corpus callosum
LatVent_excl_TH_r	Lateral ventricle (excluding temporal horn) (right)
LatVent_excl_TH_l	Lateral ventricle (excluding temporal horn) (left)
BodyVentricle_r	Lateral ventricle, body of right ventricle
BodyVentricle_l	Lateral ventricle, body of left ventricle
ThirdVentricl	Third ventricle

FL_precen_G_l	Precentral gyrus (left)
FL_precen_G_r	Precentral gyrus (right)
FL_strai_G_l	Straight gyrus (left)
FL_strai_G_r	Straight gyrus (right)
FL_OFC_AOG_l	Anterior orbital gyrus (left)
FL_OFC_AOG_r	Anterior orbital gyrus (right)
FL_i_fr_G_l	Inferior frontal gyrus (left)
FL_i_fr_G_r	Inferior frontal gyrus (right)
FL_s_fr_G_l	Superior frontal gyrus (left)
FL_s_fr_G_r	Superior frontal gyrus (right)
PL_postce_G_l	Postcentral gyrus (left)
PL_postce_G_r	Postcentral gyrus (right)
PL_s_pa_G_l	Superior parietal gyrus (left)
PL_s_pa_G_r	Superior parietal gyrus (right)
OL_ling_G_l	Lingual gyrus (left)
OL_ling_G_r	Lingual gyrus (right)
OL_cuneus_l	Cuneus (left)
OL_cuneus_r	Cuneus (right)
FL_OFC_MOG_l	Medial orbital gyrus (left)
FL_OFC_MOG_r	Medial orbital gyrus (right)
FL_OFC_LOG_l	Lateral orbital gyrus (left)
FL_OFC_LOG_r	Lateral orbital gyrus (right)
FL_OFC_POG_l	Posterior orbital gyrus (left)
FL_OFC_POG_r	Posterior orbital gyrus (right)
S_nigra_l	Substantia nigra (left)
S_nigra_r	Substantia nigra (right)
Subgen_antCing_l	Subgenual frontal cortex (left)
Subgen_antCing_r	Subgenual frontal cortex (right)
Subcall_area_l	Subcallosal area (left)
Subcall_area_r	Subcallosal area (right)
Presubgen_antCing_l	Pre-subgenual frontal cortex (left)
Presubgen_antCing_r	Pre-subgenual frontal cortex (right)
